# Decoding Subject-Driven Cognitive States from EEG Signals for Cognitive Brain–Computer Interface

**DOI:** 10.3390/brainsci14050498

**Published:** 2024-05-15

**Authors:** Dingyong Huang, Yingjie Wang, Liangwei Fan, Yang Yu, Ziyu Zhao, Pu Zeng, Kunqing Wang, Na Li, Hui Shen

**Affiliations:** 1 College of Intelligence Science and Technology, National University of Defense Technology, Changsha 410073, China; huangdingyong@nudt.edu.cn (D.H.); fanliangwei@nudt.edu.cn (L.F.); yuyangnudt@hotmail.com (Y.Y.); zhaoziyu@nudt.edu.cn (Z.Z.); zengpu@nudt.edu.cn (P.Z.); wangkunqing@nudt.edu.cn (K.W.); 2 College of Physical Education and Health, Hebei Normal University of Science & Technology, Qinhuangdao 066004, China; wangyingjie75@126.com; 3 Radiology Department, Xiangya 3rd Hospital, Central South University, Changsha 410013, China; lina2864@csu.edu.cn

**Keywords:** subject-driven cognitive states, EEG signals, time–frequency map, channel and frequency attention, brain–computer interface

## Abstract

In this study, we investigated the feasibility of using electroencephalogram (EEG) signals to differentiate between four distinct subject-driven cognitive states: resting state, narrative memory, music, and subtraction tasks. EEG data were collected from seven healthy male participants while performing these cognitive tasks, and the raw EEG signals were transformed into time–frequency maps using continuous wavelet transform. Based on these time–frequency maps, we developed a convolutional neural network model (TF-CNN-CFA) with a channel and frequency attention mechanism to automatically distinguish between these cognitive states. The experimental results demonstrated that the model achieved an average classification accuracy of 76.14% in identifying these four cognitive states, significantly outperforming traditional EEG signal processing methods and other classical image classification algorithms. Furthermore, we investigated the impact of varying lengths of EEG signals on classification performance and found that TF-CNN-CFA demonstrates consistent performance across different window lengths, indicating its strong generalization capability. This study validates the ability of EEG to differentiate higher cognitive states, which could potentially offer a novel BCI paradigm.

## 1. Introduction

Brain–computer interfaces (BCIs) are advanced technologies that establish a direct connection between the human brain and external devices [1,2,3]. BCIs can interpret users’ intentions directly from their brain signals, enabling control of external computers or devices. Currently, BCI devices have been used to assist individuals with motor disabilities to interact with the outside world, such as by enabling movement or feeling through tools like mechanical arms [4,5,6]. Based on the method of acquiring neural signals, BCI devices are mainly divided into two categories: invasive and non-invasive [7]. The invasive approach utilizes a microelectrode array, which involves surgically implanting electrodes into the depths of the brain cortex to record action potentials of individual neurons, as well as local field potentials of highly concentrated small clusters of neurons and synapses. This method provides high precision in capturing neural information, but carries a relatively high risk of damage to the human brain. In contrast, non-invasive BCIs place signal acquisition devices outside the scalp, making them more easily accepted by the general population [8]. Among non-invasive devices, EEG-based BCIs are the most commonly researched due to their low cost, ease of subject recruitment, and high temporal resolution of brain signals [9,10]. EEG-based BCIs have seen rapid development recently [11,12,13], achieving control over complex applications such as wheelchairs [14], virtual [15,16] and physical [17] quadcopters, robotic arms [18], and speech decoding [19].

In EEG-BCIs, signals can be classified into evoked and spontaneous types. Evoked EEG involves triggering specific brain responses through external stimuli, such as P300 [20,21,22,23] and Steady-State Visual Evoked Potentials (SSVEPs) [24,25,26,27,28,29]. While extensively studied in BCI systems, P300 is susceptible to interference and prolonged fixation on light sources, while SSVEPs may lead to visual fatigue. In contrast, the spontaneous motor imagery (MI) signals, generated without external stimuli, have been widely used in EEG BCIs [30,31,32,33]. Despite various advantages over existing paradigms [34], MI signals exhibit individual variability in strength and location. Users of BCIs employing MI show diverse performance levels, ranging from near-perfect control to BCI blindness (inability to meaningfully control the system) [35]. Although subjects can enhance their MI performance through learning, some may still struggle to achieve effective control even after multiple training sessions [36,37]. Challenges associated with MI include significant individual differences, high training costs, and task complexity, which impede the widespread application and advancement of this paradigm.

Some studies have initiated exploration into whether advanced cognitive activities, such as visual imagery [38] and mental arithmetic [39], could potentially serve as novel paradigms for brain–computer interfaces. However, the existing research falls short and requires further in-depth examination. Brain–computer interfaces are currently constrained by a limited range of brain signals, predominantly originating from the primary sensory–motor cortex. Further research is essential to enhance the diversity of commands in brain–computer interfaces, particularly concerning the viability of incorporating memory, reasoning, and other advanced cognitive functions as potential new paradigms for brain–computer interfaces. The feasibility of this approach and potential individual variability remain uncertain. Given the intricate and abstract nature of these advanced cognitive tasks, continued investigations are crucial to accurately capture and interpret the signals evoked by these tasks.

Functional MRI demonstrates the feasibility of distinguishing the selected advanced cognitive states (resting state, narrative memory, music, and subtraction tasks) [40]; however, the potential for differentiation using scalp EEG signals remains unexplored. Cognitive neuroscience indicates that these advanced cognitive activities involve cortical activity across the entire brain, particularly encompassing internal brain regions such as the hippocampus. EEG is considered a challenging method to capture activity from these deep brain regions, as it primarily records electrical signals from the brain’s surface cortex. Thus, distinguishing high-level cognitive activities using EEG presents a significant challenge.

In this paper, our study presents a novel methodology for effectively utilizing EEG signals to differentiate between diverse cognitive states, such as resting state, narrative memory, music, and subtraction tasks. The task is subject-dependent cognitive state detection. Firstly, through the application of continuous wavelet transform to convert raw EEG signals into time–frequency maps, we successfully extract crucial features essential for accurate classification. Secondly, the introduction of the TF-CNN-CFA model with a channel and frequency attention mechanism significantly improves the precision in distinguishing these cognitive states. Lastly, our results not only demonstrate the superior performance of the TF-CNN-CFA model over traditional methods but also highlight its consistent efficacy across varying lengths of EEG signals, underscoring its potential for practical implementation in brain–computer interface systems.

## 2. Methods

### 2.1. General Procedure

**Figure 1:**
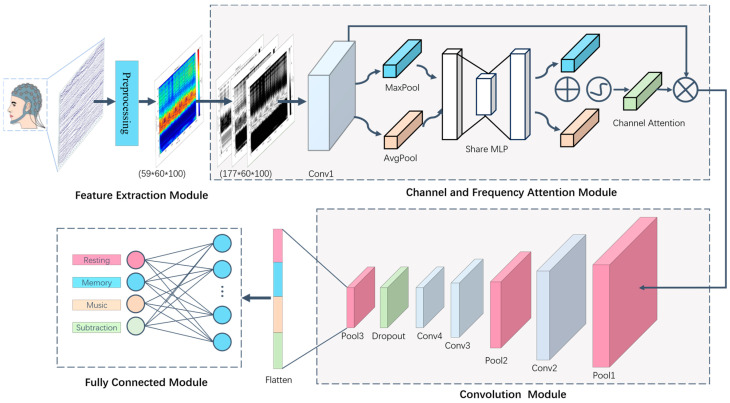
The overall framework of the proposed TF-CNN-CFA model. This network consists of a feature extraction module, a channel and frequency attention (CFA) module, a convolution module, and a fully connected module.

The overall framework of the proposed TF-CNN-CFA model is shown in Figure 1. Firstly, the raw EEG brain signals are carefully preprocessed using the EEGLAB MATLAB toolbox (MATLAB R2021b; EEGLAB v2023.1) [41], including filtering and denoising operations, to ensure high-quality data. Subsequently, we utilize continuous wavelet transform technology to transform the preprocessed EEG signals into time–frequency maps. The purpose of this step is to effectively display the characteristics of brain signals in both the time and frequency domains, thereby better revealing patterns and properties of brainwave activities. Each EEG signal channel is transformed into a time–frequency map, illustrating the activity state of this channel at different time points and frequencies. Each time–frequency map is grouped, segmented by frequency range, and decomposed into RGB channels, corresponding to frequency ranges of 0–15 Hz, 15–30 Hz, and 30–45 Hz. Next, the time–frequency maps of all channels are overlaid in the RGB dimension. Finally, the overlaid time–frequency maps are input into the network for training to decode and classify EEG signals corresponding to the four cognitive states.

We have chosen Convolutional Neural Networks (CNNs) as the primary tool for our research because CNNs exhibit significant advantages in processing image data. They can effectively analyze images and extract features from them, which makes them particularly proficient in extracting time–frequency features from EEG signals, enabling precise classification of brainwave data.

In this study, to further enhance the neural network’s processing of EEG signals, we introduce a channel and frequency attention (CFA) module. The design of this module aims to boost the network’s focus on different EEG signal channels, allowing the network to concentrate more on the feature information of key channels, thereby improving the accuracy and robustness of the classification task. By incorporating the CFA module, we anticipate strengthening the neural network’s ability to identify important channels within the EEG signals, thus enhancing the precision and efficiency of the classification process.

### 2.2. Experimental Paradigm Design

**Figure 2:**
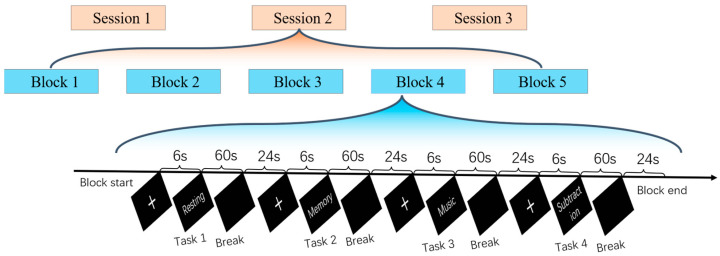
Experimental paradigm design. Each participant is required to complete three experimental sessions, each comprising 5 blocks. Within each block, participants engage in four types of mental imagery—resting, memory, music, and subtraction. In each block, a “+” signal appears on the screen to indicate the beginning of the task. Cue words for the four mental states are then displayed for 60 s, followed by a 24 s rest period. There is no break between the 5 blocks within a session, but a five-minute rest interval separates each session. At the conclusion of the experiment, each participant has 15 min of EEG data recorded for each mental imagery task state.

The experimental paradigm design is illustrated in Figure 2. Each participant is required to complete three sessions of experiments, with each session consisting of 5 blocks. In each block, the participant sequentially engages in four types of imaginations—resting, memory, music, and subtraction: a resting state, a narrative memory task, a music lyrics task, and a subtraction task. The resting state is always performed first, while the order of the three cognitive tasks is balanced. For the resting task, participants let their minds wander without focusing on anything specific. For the memory task, participants are asked to recall events from the moment they woke up until the current time. In the music task, participants are instructed to mentally sing their favorite song lyrics. In the subtraction task, participants are required to count down from 5000 in increments of 3. Participants are instructed to maintain an open-eyed state in a self-driven cognitive state throughout.

In each block, a 6 s display of “+” on the screen signals the participant to prepare for the experiment, during which the participant is required to gaze at the center of the screen where the “+” is located without moving their body. Subsequently, cue words indicating the four states are displayed on the screen, prompting the participant to use their imagination for 60 s. During this period, the participant must concentrate, avoid head and body movements, and minimize blinking. Finally, a 24 s rest period follows, allowing the participant to relax with minimal body movement as the screen remains blank. A one-minute rest interval separates two consecutive blocks, while a five-minute break is provided between sessions. At the end of the experiment, each participant has 15 min of EEG experimental data for each task state.

**Figure 3:**
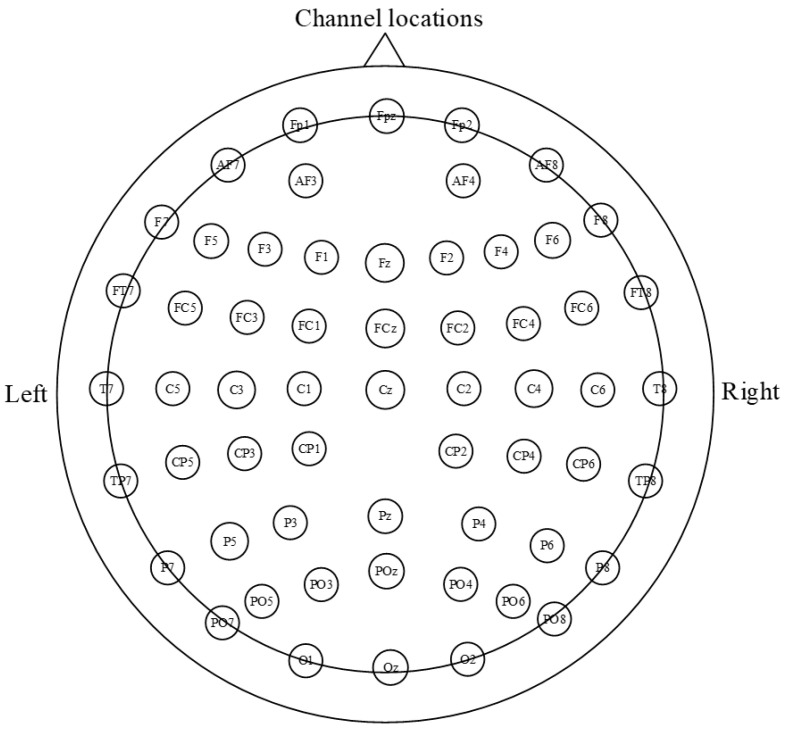
Electrode distribution (59 electrodes placed according to the international 10–20 system).

In this study, the EEG data were acquired with a Neuracle EEG amplifier at a sampling rate of 1000 Hz. The 59 electrodes were placed according to the international 10–20 system at positions including Fpz, Fp1, Fp2, AF3, AF4, AF7, AF8, Fz, F1, F2, F3, F4, F5, F6, F7, F8, FCz, FC1, FC2, FC3, FC4, FC5, FC6, FT7, FT8, Cz, C1, C2, C3, C4, C5, C6, T7, T8, CP1, CP2, CP3, CP4, CP5, CP6, TP7, TP8, Pz, P3, P4, P5, P6, P7, P8, POz, PO3, PO4, PO5, PO6, PO7, PO8, Oz, O1, and O2. This electrode arrangement aims to comprehensively cover different brain regions for comprehensive and accurate recording of brainwave activity, as depicted in Figure 3.

Seven healthy male participants aged 22 to 28 years took part in the experiment, all with normal vision or corrected vision with glasses, and no history of electrical or drug therapy 30 days prior to the experiment. They were all right-handed and had no mental illnesses. Participants refrained from staying up late the night before the experiment, ensuring good rest, and abstained from alcohol, smoking, and tea consumption. Detailed explanations of the experimental tasks and procedures were provided to participants before the experiment. All participants signed written informed consent forms and were involved in the experiment. This study was conducted in accordance with the Declaration of Helsinki and approved by the Ethics Committee of the Xiangya Hospital of Central South University.

During the data collection process, participants sat comfortably in a chair 0.5 m away from the monitor, with their hands naturally resting on the armrests. The experiments were conducted in a quiet, well-lit, and temperature-controlled laboratory environment. Before the formal experiment commenced, participants were required to complete a 30 min training task. The training session aimed to familiarize participants with the experimental tasks and procedures by practicing each of the four cognitive states—resting, memory, music, and subtraction—five times.

### 2.3. Data Preprocessing

**Figure 4:**
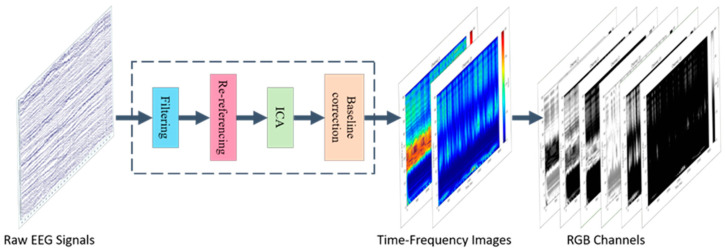
Data preprocessing flowchart. The data preprocessing procedure involved band-pass filtering (0.1–45 Hz), the average reference method, Independent Component Analysis (ICA), interpolation repair, data segmentation, and baseline correction. Subsequently, continuous wavelet transform was used to create time–frequency images, which were organized by frequency range and decomposed into RGB channels representing frequency ranges of 0–15 Hz, 15–30 Hz, and 30–45 Hz.

In our study, we employed the EEGLAB toolbox in MATLAB to preprocess the raw EEG signals. The data preprocessing workflow, as illustrated in Figure 4, involves several key steps. Initially, we applied band-pass filtering (0.1–45 Hz) to the collected EEG signals to retain information within the specific frequency range of interest while reducing noise interference, thereby enhancing signal quality for clearer analysis and interpretation. Due to the high number and even distribution of electrodes on the cap used, we utilized the average reference method to obtain a reference electrode and performed offline re-referencing of the EEG data. Subsequently, the EEG data were decomposed into independent components using Independent Component Analysis (ICA). By identifying components with artifacts related to eye movements, heartbeats, muscle activities, and other non-neural activity-related artifacts, these components were removed to preserve authentic brainwave signals. The data were then reorganized to eliminate artifact effects and retain genuine brainwave signals. For bad channels, interpolation repair using the commonly used spherical spline method was conducted based on data from normal channels. Following this, the data were segmented according to the experimental task labels for the four tasks (resting, memory, music, and subtraction), with an additional baseline correction involving the extraction of 6000 ms of resting time before the start of each task.

Each participant provided 60 min of EEG data, with each of the four states having an interval of 15 min. To further analyze the data, we segmented the EEG data for each state into three-second intervals without overlap. Subsequently, we utilized continuous wavelet transform technology to generate corresponding color-coded time–frequency maps. These maps display changes in signal energy intensity across different frequencies over time, with the *X*-axis representing time (0–3000 ms) and the *Y*-axis representing frequency (0–45 Hz). The color mapping in time–frequency images, achieved through continuous wavelet transform (CWT), presents an alternative visualization technique for illustrating the distribution of signal energy across various frequencies. Warm hues, such as red and orange, signify high signal power, whereas cooler tones like blue and purple indicate low power levels, effectively capturing temporal and spectral fluctuations in signal intensity. Nonetheless, it is crucial to recognize that this color-coded depiction constitutes an esthetic variation; fundamentally, the information conveyed is consistent with that of grayscale time–frequency images, differing only in visual presentation.

These time–frequency images were grouped, segmented by frequency range, and decomposed into RGB channels, corresponding to frequency ranges of 0–15 Hz, 15–30 Hz, and 30–45 Hz. Each channel shows the energy distribution within different frequency ranges: the red channel corresponds to low-frequency energy, the green channel to moderate frequency energy, and the blue channel to high-frequency energy. The magnitude of grayscale values reflects the power within each frequency band, with higher values indicating higher power. By overlaying the grayscale values from all three channels, the original colored image was obtained, showcasing the signal strength across all frequencies.

After compressing each single-channel time–frequency map to a size of 60 × 100, we combined the 59 channels’ time–frequency maps onto the RGB channels to create composite images with 177 channels (59 RGB three-channel overlays). Finally, these composite images were used as input data for the neural network training, with a dimension of (32, 177, 60, 100)—where 32 denotes batch size, 177 represents the number of channels in the time–frequency maps, 60 signifies the height of the time–frequency maps, and 100 indicates the width of the time–frequency maps. These processed data were subsequently fed into the neural network for training.

### 2.4. Network Architecture

In this study, we propose a custom convolutional neural network model named TF-CNN-CFA designed to handle EEG signal classification tasks. The neural network model comprises several key components, including convolutional layers, pooling layers, and fully connected layers. Specifically, the model consists of four convolutional layers (Conv1, Conv2, Conv3, and Conv4), with Conv4 incorporating a dropout layer and an ReLU non-linear activation function layer to enhance model generalization and non-linear feature representation. Additionally, the model includes three pooling layers (Pool1, Pool2, and Pool3) to reduce feature dimensions and extract salient features. Notably, the model integrates a channel and frequency attention (CFA) module aimed at enhancing the network’s focus on different EEG signal channels to capture crucial features and improve classification accuracy. Finally, the model structure encompasses a fully connected layer (FC) responsible for learning feature representations and achieving the 4-classification task. The network parameters are detailed in Table 1.

The input layer N1 is sized at (32, 177, 60, 100). It undergoes a convolutional operation with a 3 × 3 kernel size (conv1) to produce N2, maintaining the feature map size at (32, 177, 60, 100). Subsequently, a CFA module operation is applied to maintain the size. Next, a 5 × 5 pooling operation (pool) is performed on N2 to obtain N3, resulting in a feature map size of (32, 177, 12, 20). Following this, a 5 × 5 convolution operation with conv2 on N3 generates N4 with a feature map size of (32, 128, 12, 20). The subsequent 2 × 2 pooling operation (pool2) on N4 yields N5 with a feature map size of (32, 128, 6, 10). N5 then undergoes a 5 × 5 convolution operation (conv3) to produce N6 with a feature map size of (32, 128, 6, 10). This is followed by another 5 × 5 convolution operation (conv4) on N6 to obtain N7 with a feature map size of (32, 64, 6, 10). A dropout operation is conducted on N7, maintaining its size before a 2 × 2 pooling operation (pool4) on N7 produces N8 with a feature map size of (32, 64, 3, 5). N8 is then flattened into a one-dimensional vector to obtain N9 with a size of (32, 960). Finally, a fully connected layer (fc) maps N9 to N10, achieving the 4-classification task.

#### 2.4.1. Channel and Frequency Attention (CFA) Module

In our study, we introduce a CFA module to enhance the neural network’s performance of classifying EEG signals. This module receives an input tensor of dimensions (32, 177, 60, 100), where the batch size is 32, the number of channels in the time–frequency maps is 177 (derived from 59 RGB three-channel time–frequency maps), and the height and width of the time–frequency maps are 60 and 100, respectively. This module allows for dynamic adjustments of weights across different channels, enabling the network to focus more on key channel information to enhance model performance and accuracy.

The specific operational flow is as follows: Initially, through global max-pooling and global average-pooling operations, features of each channel are extracted to a spatial dimension of 1 × 1, resulting in an output size of (32, 177, 1, 1). Subsequently, a fully connected layer (fc1) reduces the number of channels to 59, applying ReLU activation for non-linear transformation. Then, another fully connected layer (fc2) restores the number of channels to 177, followed by another application of ReLU activation. Finally, channel weights are normalized using the Sigmoid activation function, multiplying the channel weights with the input feature map to produce an output feature map with a size of (32, 177, 60, 100). The network structure of the CFA module is depicted in Table 2.

By integrating the CFA module, the neural network can better learn and utilize key channel features, enhancing focus on important channels, thereby improving model performance and accuracy when processing EEG signals. The introduction of this module significantly enhances and optimizes EEG signal classification tasks.

#### 2.4.2. Training Process and Strategies

To ensure the stability of the model, we employed a five-fold cross-validation method during training. Within the limited dataset context, each participant’s data samples (1200) were divided into an 80% training set (960) and a 20% test set (240). Subsequently, the 960 training samples per participant were randomly split into 5 equal parts. Four of these parts were assigned to the training set, while the remaining fifth part was used as the validation set for model verification. Following the completion of model training, testing was conducted using the test set to obtain classification accuracy for each fold. Finally, the average accuracy across the five folds was calculated and considered the model’s classification outcome.

In the training process, we utilized the Adam optimizer, a commonly preferred optimization algorithm known for its simplicity, high computational efficiency, and minimal memory usage. With bias correction applied, the optimizer ensured that the learning rate for each iteration remained within a specific range, contributing to parameter stability. The chosen loss function was the cross-entropy loss function, well-suited for multi-classification networks, as depicted in Equation (1). Cross-entropy serves to measure the disparity between two different probability distributions within the same random variable, representing the difference between the actual probability distribution and the predicted probability distribution.

(1) $$\mathrm{Loss}\;=-\sum p_{i}(x)logq_{i}(x),i=1,2,\cdots ,n$$

where x represents a single data sample, n denotes the total number of categories, indicating how many categories need to be classified, $p_{i}(x)$ represents the i-th target probability distribution, and $q_{i}(x)$ denotes the i-th predicted probability distribution. A smaller cross-entropy loss value indicates improved predictive performance of the model.

The training configuration involved 50 epochs with a batch size of 32 for each iteration. To achieve superior training outcomes, the network also implemented the dropout technique with a probability of P = 0.5 to mitigate overfitting and gradient explosions. Additionally, the ReLU activation function was utilized as part of the network architecture to introduce non-linearity and address vanishing gradient issues effectively, as demonstrated in Equation (2).

(2) $$\mathrm{ReLU}\left(x\right)=\left\{\begin{array}{ll} x & \mathrm{if}\;x>0\\ 0 & \mathrm{if}\;x\leq 0 \end{array}\right.$$

#### 2.4.3. Evaluation Metrics

In assessing model performance, we utilize various metrics including accuracy (*Acc*), precision (*P*), recall (*R*), *F*-score, and Kappa coefficient ($P_{k}$).

Accuracy (*Acc*): Accuracy represents the proportion of correctly classified samples over the total number of samples in the test set. It is calculated as shown in Equation (3):

(3) $$Acc=\frac{TP+TN}{TP+TN+FP+FN}$$

where *TP* denotes true positives (correctly predicted positive instances), *TN* represents true negatives (correctly predicted negative instances), *FP* signifies false positives (negative instances incorrectly predicted as positive), and *FN* indicates false negatives (positive instances erroneously predicted as negative).

Precision (*P*) and Recall (*R*): Precision measures the ratio of correctly classified positive samples to the total samples classified as positive, while recall evaluates the ratio of correctly classified positive samples to all actual positive samples. The formulas are given by the following:

(4) $$P=\frac{TP}{TP+FP},R=\frac{TP}{TP+FN}$$

*F*-score: The *F*-score combines precision and recall, providing a single metric that balances both aspects. It is computed as shown in Equation (5):

(5) $$F\text{-}\mathrm{score}\;=\frac{2\times R}{P+R}$$

Kappa Coefficient ($P_{k}$): The Kappa coefficient is utilized for consistency testing and to gauge classification accuracy. It is expressed as follows in Equation (6):

(6) $$P_{k}=\frac{Acc-P_{0}}{1-P_{0}}$$

Here, $P_{0}$ represents the chance-corrected agreement.

## 3. Results

### 3.1. Model Performance Analysis

We evaluated the overall performance of the model, with EEG signal time lengths *L* set to 3 s, using five-fold cross-validation, and conducting experiments on a self-collected dataset. The experimental results are presented in Table 3.

Overall, the experimental outcomes demonstrate high accuracy and a certain level of consistency. The average accuracy stands at 76.14%, with a standard deviation of 9.13%, indicating some variability across different participants.

Observing the results for the seven participants, except for S3 and S5, the accuracy rates surpass 70% for the remaining individuals. Notably, S1 and S7 exhibit exceptional accuracy rates of 86.61% and 90.89%, respectively, marking them as the top-performing participants in the experiment. Their average accuracy rates both exceed 85%, showcasing the model’s outstanding recognition capabilities for specific individuals.

However, some participants exhibit a more moderate performance. For instance, S2, S3, S4, and S5 achieve accuracy rates of 73.04%, 66.70%, 71.16%, and 69.20%, respectively. Particularly concerning is S3, with an accuracy rate of only 66.70%. Moreover, the precision, recall, F1 score, and Kappa coefficient for S3 are relatively lower, at 73.09%, 66.70%, 65.55%, and 55.69%, respectively, indicating a degree of fluctuation in model performance across participants.

**Figure 5:**
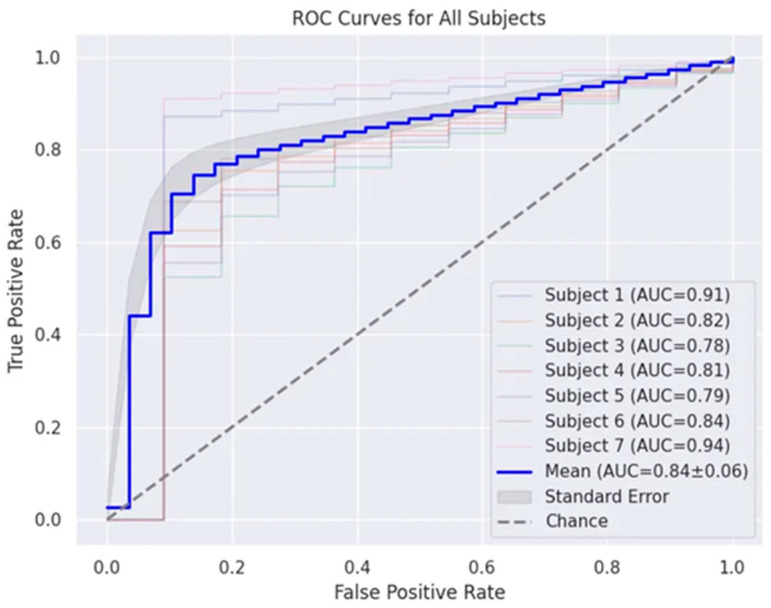
ROC curves. The horizontal axis represents the false positive rate (FPR), while the vertical axis represents the true positive rate (TPR). Each participant has an individual ROC curve, with the thick blue line depicting the average ROC curve across all participants. The shaded area indicates the standard deviation. The diagonal line represents the random classification probability.

In order to gain a deeper understanding of each participant’s classification performance, we generated ROC curves for the seven participants, as displayed in Figure 5.

The results indicate that participant 7 exhibited outstanding performance, with an AUC value as high as 0.94. This signifies the model’s exceptional ability in distinguishing between the four different states, demonstrating strong category discrimination capability. Participant 1 also achieved notable results, obtaining an AUC value of 0.91, further confirming the model’s outstanding performance. However, participant 3 showcased the lowest AUC value at 0.78, highlighting fluctuations in classification performance across different participants.

The overall average AUC of 0.84 with a standard deviation of 0.06 suggests a relatively stable classification performance across the varied participants. This implies that the model performs well in multi-classification tasks, displaying good overall performance despite minor individual differences. Considering the collective performances of all participants, it is evident that the model exhibits effectiveness and robustness in recognizing the states of resting, memory, music, and subtraction.

**Figure 6:**
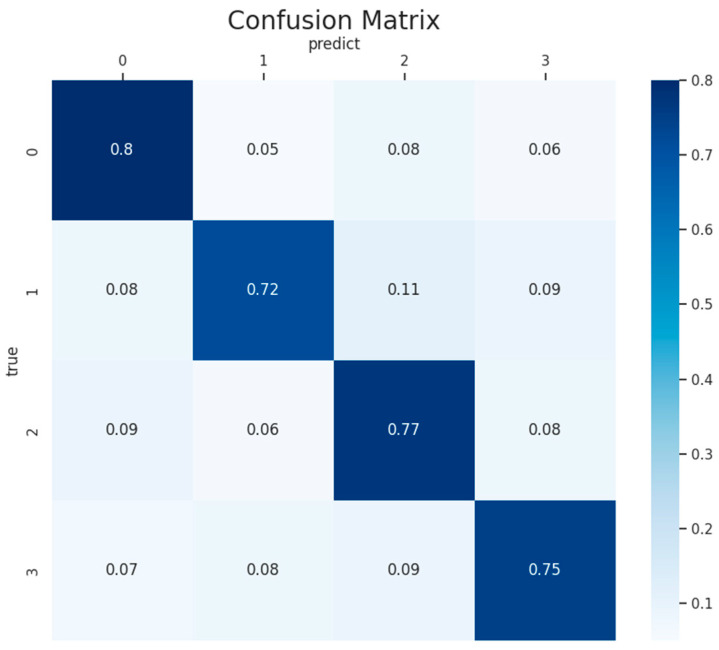
An average confusion matrix of the 7 participants using the TF-CNN-CFA network model on the self-collected dataset. The horizontal axis represents predicted labels, while the vertical axis denotes true labels. The numbers “0”, “1”, “2”, and “3” represent “Resting”, “Memory”, “Music”, and “Subtraction”, respectively. The element (i, j) indicates the probability of samples of class i being classified as class j.

To assess the recognition capabilities for each state, we present the average confusion matrix for all participants in Figure 6. It is observed that the “Resting” state is relatively easier to identify compared to “Memory”, “Music”, and “Subtraction”, boasting the highest classification accuracy of 80%. Additionally, the classification accuracies for “Resting”, “Memory”, “Music”, and “Subtraction” all exceed 72%. This indicates that the classification accuracies for the four categories are quite close, suggesting good model performance, with effective classification results for each category.

The algorithm demonstrates a low false positive rate for the “Resting” state, indicating its ability to correctly identify instances of this state. In the case of the “Memory” and “Music” states, the algorithm shows low misclassification rates, with both low false positives and false negatives, highlighting its accuracy in differentiating these states. Conversely, the higher false positive rates for the “Subtraction” state suggest that there is room for improvement in accurately classifying this state.

However, the classification accuracy for the “Memory” state is relatively lower, with probabilities of misclassification as “Resting”, “Music”, and “Subtraction” standing at 8%, 11%, and 9%, respectively. This phenomenon may be attributed to the complexity and diversity inherent in memory processes. Memory involves various cognitive processes and brain regions, presenting differences in brain activity patterns, potentially leading to increased difficulty in identification. From a physiological perspective, memory entails the coordinated action of multiple brain regions, such as the hippocampus and frontal cortex, contributing to its complexity and potentially resulting in diverse and challenging-to-capture features of memory states within EEG signals.

#### 3.1.1. Ablation Analysis

**Figure 7:**
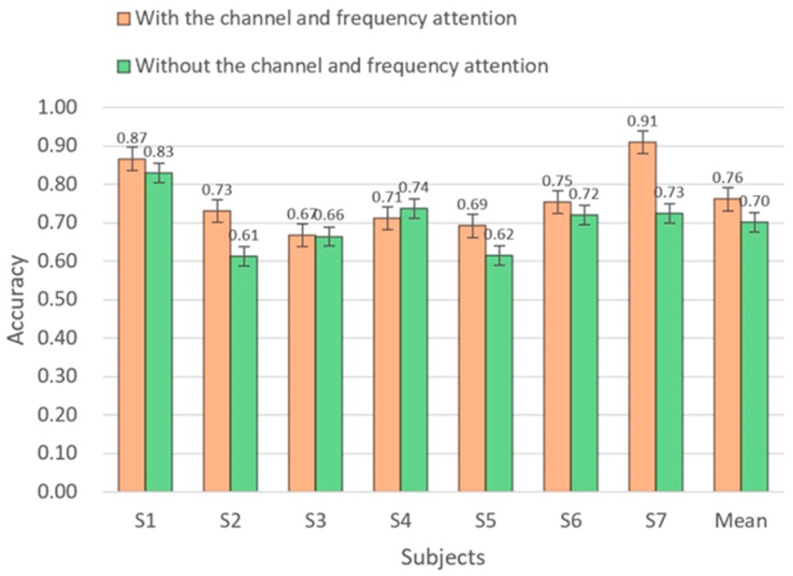
Channel and frequency attention (CFA) module performance analysis. The horizontal axis represents participants, while the vertical axis represents accuracy.

This study proposed a channel and frequency attention (CFA) module aimed at extracting the channels and frequencies that contribute significantly to the classification task, enhancing the classification performance of these four major subject-driven higher cognitive states. The experimental results are illustrated in Figure 7.

The experimental outcomes reveal that incorporating the CFA module led to an overall enhancement in the model’s classification accuracy. With the CFA module in place, the average classification accuracy across participants was 0.7614, compared to 0.7005 without the CFA module. This indicates that the CFA module played a beneficial role in improving the overall classification accuracy. Particularly noteworthy is the significant improvement in classification performance observed in participant 2 and participant 7, who initially exhibited poorer classification results. Thus, the results of this study suggest that introducing the CFA module effectively boosts the classification accuracy of EEG signals.

**Figure 8:**
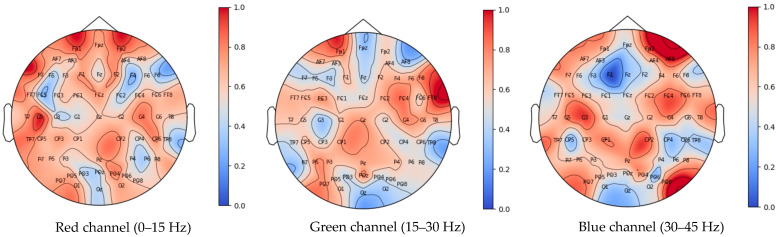
Brain channel and frequency attention weight distribution map for the seven participants (normalized). Blue indicates weak brainwave signals in the area, while darker shades of red indicate stronger brainwave signals in that region.

Moreover, the proposed CFA module also contributes to enhancing the model’s interpretability and visualization. The CFA module can highlight the importance weights of each signal channel in different state classifications, making the model’s decision-making process more transparent and understandable. Figure 8 illustrates the distribution of the average channel and frequency attention weight coefficients for the seven participants on brain topographic maps. The present study found that channels such as C5, FP1, FP2, C4, PO4, TP7, CP6, T7, F1, and F2 in the low frequency range (0–15 Hz), channels including FT8, FC4, FP1, F8, C4, PO7, POz, P5, FC3, and CP1 in the mid-frequency range (15–30 Hz), as well as channels like AF8, C3, Fp2, FC4, CP2, PO8, C5, CP1, FC6, C4, and PO7 in the high frequency range (30–45 Hz) play a significant role in the classification of the four cognitive states.

#### 3.1.2. Qualitative Analysis (Visual Analysis)

Following a series of quantitative evaluation tasks, we conducted a qualitative assessment using T-distributed Stochastic Neighbor Embedding (T-SNE) [42] to evaluate the discriminative capabilities of feature vectors. T-SNE is widely used for projecting high-dimensional data onto a two-dimensional scatter plot. All experiments in this study were based on our self-collected dataset, conducting individual participant classification experiments under the same training strategy.

**Figure 9:**
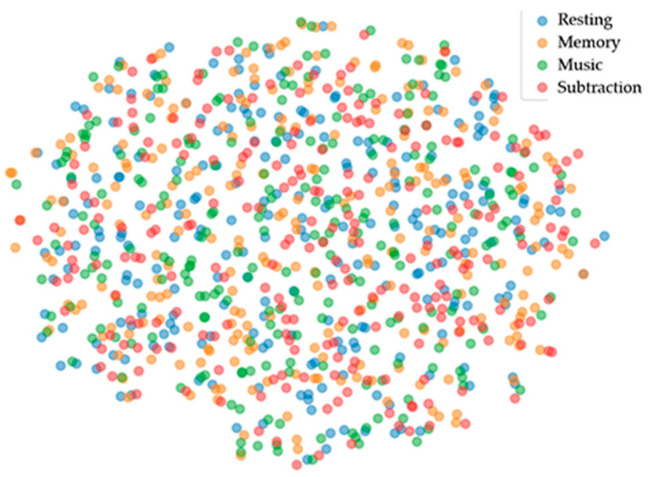
T-SNE mapping of participant 7’s original data.

**Figure 10:**
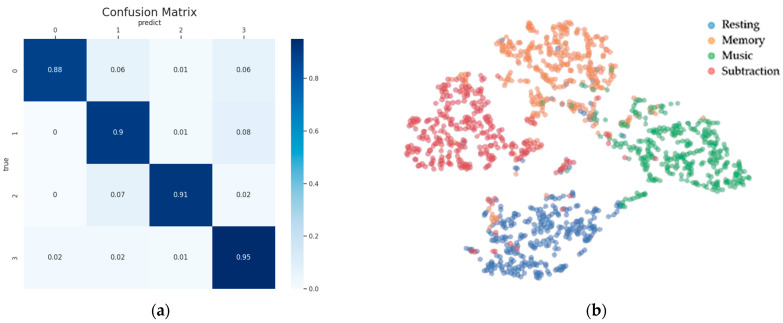
Confusion matrix and corresponding T-SNE mapping of participant 7 after classification. The horizontal axis of the confusion matrix represents predicted labels, while the vertical axis denotes true labels. The numbers “0”, “1”, “2”, and “3” represent “Resting”, “Memory”, “Music”, and “Subtraction”, respectively. (**a**) Confusion matrix of participant 7 after classification. (**b**) Corresponding T-SNE mapping of participant 7 after classification.

In Figure 9, the labels of all categories of participant 7’s original EEG data are evenly distributed in the T-SNE plot. Different colors represent the labels of EEG signals for the four brain states (resting, memory, music, and subtraction). The confusion matrix of participant 7 after classification and its corresponding T-SNE plot are presented in Figure 10. It is observed that the T-SNE plot and confusion matrix exhibit similar trends. Under high classification accuracy, the T-SNE scatter plot shows closer clustering within the same category and clearer separation between different categories. When misclassifications occur, there may be some overlap between the scatter points of different categories in the T-SNE plot.

The t-SNE visualization effectively demonstrates the classification performance, showcasing the superior classification results of the TF-CNN-CFA network model in distinguishing between the four different brain states. This further confirms the model’s effectiveness in multi-class classification tasks, providing compelling visual evidence and guiding directions for future model improvements and optimizations.

### 3.2. Comparative Analysis

We compared the TF-CNN-CFA with EEGNet_4_2 [43], EEGNet_8_2 [43], EEGNex [44], ResNet18 [45], VGG16 [46], LeNet [47], and TF-CNN. EEGNet_4_2, EEGNet_8_2, and EEGNex take raw signals as input, while TF-ResNet18, TF-VGG16, TF-LeNet, TF-CNN, and TF-CNN-CFA take time–frequency maps as input after feature representation. The comparison results of the accuracy, precision, recall, F-score, Kappa, and AUC values for different models on the 3000 ms data are presented in Table 4.

The results demonstrate that the TF-CNN-CFA model exhibits significant advantages in multiple key performance metrics. Firstly, its accuracy reaches 0.76 ± 0.09, notably higher than that of the other models, indicating outstanding performance in correctly predicting sample categories. Secondly, the model’s precision of 0.79 ± 0.07 surpasses that of other models by far, signifying a high proportion of true positive instances among all predicted positive instances.

Furthermore, in terms of recall, TF-CNN-CFA also demonstrates excellent performance at 0.76 ± 0.09, indicating successful identification of most actual positive instances. Considering the F1 score, which balances precision and recall, TF-CNN-CFA achieves a score of 0.76 ± 0.10, showcasing good balanced performance in classification tasks. Additionally, the Kappa coefficient of 0.68 ± 0.12 reveals significant consistency between the model’s predictions and random selection.

Lastly, in the AUC value aspect, TF-CNN-CFA achieves a score of 0.84 ± 0.06, demonstrating a large area under the ROC curve and overall superior performance. In conclusion, based on a comprehensive comparative analysis of the models across performance metrics, it is evident that the TF-CNN-CFA model excels on the dataset, displaying strong classification and generalization abilities. Therefore, it can be concluded that the TF-CNN-CFA model performs remarkably well in this task and is a deep learning model worthy of further research and application.

Further comparison of the performance of the top four neural network architectures on our self-collected dataset, including EEGNex, TF-LeNet, TF-CNN, and TF-CNN-CFA, was conducted. In Table 5, a detailed analysis of the accuracy (Acc) and Kappa coefficient values of these models across different participants is provided.

The results show that the TF-CNN-CFA model excels across the entire dataset, with an average accuracy of 0.7614, significantly higher compared to the other models. Particularly noteworthy is the exceptional performance of the TF-CNN-CFA model in the testing of participant S7, achieving an accuracy of 0.9089 and a Kappa coefficient of 0.8784, highlighting its outstanding performance on this specific participant. This indicates the robust and reliable overall performance of the TF-CNN-CFA model on the dataset. Furthermore, the TF-CNN model exhibits excellent performance on participants S1 and S4, with accuracy rates of 0.8295 and 0.7366, respectively, and Kappa coefficients of 0.7724 and 0.6491. This demonstrates the standout performance of the TF-CNN model on specific participants and its decent generalization capabilities. While the TF-LeNet and EEGNex models also show relatively good performance on some participants, their overall performance slightly lags behind that of the TF-CNN-CFA and TF-CNN models.

Considering the overall average values, the TF-CNN-CFA model significantly outperforms the others in terms of accuracy and Kappa coefficients, reaching values of 0.7614 and 0.6818, respectively. Through an analysis of standard deviations, it is revealed that the performance of the TF-CNN-CFA model exhibits low variance, indicating high stability and reliability on the dataset. This suggests that the TF-CNN-CFA model not only excels on individual participants but also possesses excellent generalization capabilities across the dataset.

**Figure 11:**
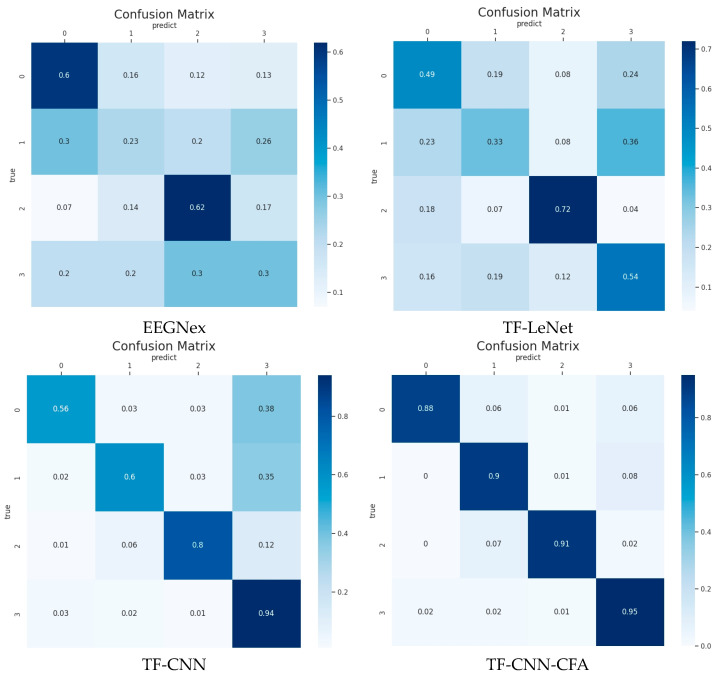
Confusion matrix for participant 7 with the different models. The vertical axis represents the true labels of the four cognitive state categories, while the horizontal axis indicates the predicted labels of the four cognitive state categories. “0”, “1”, “2”, and “3” correspond to “Resting”, “Memory”, “Music”, and “Subtraction”, respectively. The element (i, j) represents the probability of a sample belonging to class j within the i-th class.

To analyze the effects of the model proposed in this study on EEG data recognition for each state, we computed the average confusion matrix for participant 7 across four different models. As depicted in Figure 11, the horizontal axis represents the predicted categories of EEG states by the models, while the vertical axis denotes the actual EEG state categories. The values on the diagonal represent the proportion of correct classifications, while the off-diagonal elements indicate the proportion of misclassifications.

In this study, we combined an attention module with a CNN to process EEG data. By extracting features from a global perspective and generating channel and frequency attention maps, we aimed to reduce errors in the classification of the four brain states. For participant 7, the accuracy rates for the four states reached 88%, 90%, 91%, and 95%, respectively. Compared to the EEGNex, TF-LeNet, and TF-CNN models, the TF-CNN-CFA model demonstrated superior performance. Additionally, the minimal differences in classification accuracy among different categories suggest that the model exhibits stable and excellent performance.

### 3.3. Time Length Impact

In the realm of brain–computer interface (BCI), a crucial issue is the reduction in the EEG signal length *L* to obtain sufficient information for robust classification. In other words, the aim is to achieve high classification accuracy while using shorter EEG signal lengths.

In this respect, we also performed classification tasks using EEG signals of different time lengths: *L* = 384 (1.5 s), 512 (2 s), 640 (2.5 s), 768 (3 s), 896 (3.5 s), 1024 (4 s), 1152 (4.5 s), and 1280 (5 s). The size of each dataset varies accordingly. Table 6 presents the number of training samples and testing samples as shown below. Following the established procedure, we trained the network through 5-fold cross-validation with 20 epochs per fold. Subsequently, we compared the results obtained with this approach to EEGNet_4_2, EEGNet_8_2, EEGNex, TF-ResNet18, TF-VGG16, TF-LeNet, and TF-CNN methods.

Now, we consider comparing the classification performance of EEG signals of different time lengths *L*. We compare TF-LeNet, TF-CNN, and our network model TF-CNN-CFA. Table 7 presents the performance results for time lengths of 1.5 s, 2 s, 2.5 s, 3 s, 3.5 s, 4 s, 4.5 s, and 5 s. These results correspond to the average validation accuracy obtained from K-fold cross-validation (K = 5) for each category within the four classes and the overall average accuracy.

Firstly, in terms of overall accuracy, TF-CNN-CFA outperformed all models across all time lengths. In particular, at time lengths of 1.5 s and 2 s, its overall accuracy reached 0.87 and 0.83, respectively, significantly surpassing that of TF-LeNet and TF-CNN. This highlights the outstanding performance of TF-CNN-CFA in processing EEG data over short time lengths *L*.

Secondly, as the time length *L* increased, the performance of all models showed a decreasing trend. However, even at longer time lengths, TF-CNN-CFA maintained a relatively high accuracy, demonstrating its exceptional capability in handling long time length data.

Compared to TF-LeNet and TF-CNN, TF-CNN-CFA not only led in overall accuracy but also excelled in performance in each category. At each time length *L*, TF-CNN-CFA achieved higher classification accuracies, particularly excelling in categories 1 and 3.

From a generalization perspective, while the performance of all models decreased with increasing time length *L*, TF-CNN-CFA exhibited a more stable performance across different time lengths *L*. This indicates that TF-CNN-CFA possesses good generalization ability and can effectively handle EEG data of varying lengths.

## 4. Discussion

In this study, we proposed an EEG experimental paradigm involving four different cognitive states, including resting, narrative memory, music, and subtraction tasks for potential cognitive BCI applications. The collected raw EEG signals were transformed into time–frequency maps through continuous wavelet transform and a convolutional neural network model TF-CNN-CFA with a channel and frequency attention mechanism was designed to classify these cognitive states. The experimental results showed that the average classification accuracy of the seven participants across the four cognitive states reached 76.14% ± 0.09, significantly outperforming several comparative methods.

This study introduces a deep learning method based on time–frequency maps that effectively distinguishes between these four states, validating that advanced cognitive activities can serve as a potential paradigm for brain–computer interfaces. This paradigm offers several advantages. Firstly, it is simple and easy to implement, reducing the impact of individual differences on the results. Participants do not require extensive training to acquire the skills, thus saving time and costs. Secondly, the diverse and engaging design of the four states alleviates cognitive load, enhancing operability and efficiency. This article represents the first attempt at EEG decoding based on high-level cognitive activities, covering various cognitive domains such as auditory processing, memory, and reasoning. It confirms the feasibility of utilizing advanced cognitive activities in BCIs. This study aims to translate brain thoughts into commands, thereby expanding the range of instructions available for brain–machine interfaces.

The TF-CNN-CFA model proposed in this study significantly enhances the classification performance of BCI tasks. According to the data in Table 4, direct classification of raw EEG brain signals yielded poor results, with classification accuracies of 0.27 ± 0.02 for EEGNet_4_2, 0.26 ± 0.03 for EEGNet_8_2, and 0.36 ± 0.08 for EEGNex. To address this issue, the raw EEG signals were transformed into time–frequency maps through continuous wavelet transform. These time–frequency maps were then grouped and segmented based on frequency ranges and decomposed into RGB images with three channels corresponding to frequency ranges of 0–15 Hz, 15–30 Hz, and 30–45 Hz. Each channel displayed the energy distribution within different frequency ranges. The time–frequency maps were subsequently fed into the network for training. Table 4 demonstrates an improvement in classification performance after transforming the signals into time–frequency maps, with average classification accuracies of 0.43 ± 0.08 for TF-LeNet, 0.70 ± 0.08 for TF-CNN, and 0.76 ± 0.09 for TF-CNN-CFA models. The conversion of data into time–frequency maps is essential when dealing with advanced cognitive tasks. The experimental findings suggest that advanced cognitive activities may engage brainwave signals across various frequency ranges throughout the entire brain, making the use of raw EEG signals ineffective. These activities are more intricate than simple imagination, rendering conventional EEG signal processing methods unsuitable. Furthermore, the TF-CNN-CFA model showed minimal differences in classification accuracy among different categories, indicating its stable and excellent performance. By introducing CFA modules, the model’s classification performance was significantly enhanced. As shown in Figure 8, after incorporating these attention modules, the average classification accuracy for all subjects increased from 70.05% to 76.14%. Notably, subject 2 and subject 7 saw improvements in classification accuracy by 11.69% and 18.39%, respectively, after processing with the CFA modules.

Despite our relatively good results, there are some limitations that need further discussion. First, we can learn from the research approach of Veeranki et al. [48], who effectively utilized non-linear signal processing for emotion detection via EDA. Adopting this non-linear-focused approach can refine our EEG-based BCI system and enhance its ability to decipher complex cognitive processes. Second, the multi-layer architecture and attention mechanism do help it to capture relevant features efficiently, but this also leads to an increase in the number of parameters, which increases the computational cost and training time. Third, with the increased complexity, the algorithm faces challenges in convergence during training. Optimizing such a network requires careful tuning of hyperparameters, including learning rates, dropout rates, and the number of epochs, which can be a painstaking and computationally expensive process. Fourth, the limited sample size in this study may lead to overfitting, which could impact the model’s ability to generalize to unseen EEG data. Further research is underway to investigate cognitive state classification on larger-scale datasets. Fifth, the classifier performance is heavily dependent on the EEG signal denoising effect, and the commonly used ICA method for preprocessing is used in this paper. Some more robust preprocessing denoising methods have been proposed in recent years [49,50,51,52,53] which may further enhance the classification effect. We will further evaluate the effect of EEG noise on classification performance in future studies. In addition, cognitive activity is heavily subject-dependent and varies widely between subjects. Cognitive BCI stable feature representation is still a direction for further exploration in the future. Interest in cognitive BCI has grown in recent years. Vansteensel et al. [54] documented the successful manipulation of a computer cursor toward a target through the modulation of gamma electroencephalography (ECoG) activity within the left dorsolateral prefrontal cortex (DLPFC). Ryun et al. [55] demonstrated the predictability of two distinct motor actions (hand grasping and elbow bending) prior to their execution, achieved by analyzing prefrontal ECoG signals. These invasive investigations collectively illuminated the neural mechanisms supporting cognitive BCIs and bolstered the practical potential of this technology. Simultaneously, endeavors to create non-intrusive cognitive BCIs in humans, employing electroencephalography (EEG) [56,57] and near-infrared spectroscopy (NIRS) methodologies [58], have gained momentum. Thereby validating the practicality of non-invasive cognitive BCI systems.

## 5. Conclusions

This study explores a novel EEG-BCI paradigm involving four subject-driven cognitive state tasks: resting, narrative memory, music, and subtraction. By employing the TF-CNN-CFA model with a channel and frequency attention module to classify these four cognitive states, the results demonstrate an average classification accuracy of 76.14% across seven participants, significantly outperforming other classification methods of EEG-BCI. This study can also enrich cognitive brain–machine interface paradigms.

## Figures and Tables

**Table 1:** Specific network structure.

Input	Layer	Output	Feature Maps	Kernel	Stride
N1(32, 177, 60, 100)	conv1	N2(32, 177, 60, 100)	177	3 × 3	1
N2(32, 177, 60, 100)	CFA module	N2(32, 177, 60, 100)	177	-	-
N2(32, 177, 60, 100)	pool1	N3(32, 177, 12, 20)	177	5 × 5	5
N3(32, 177, 12, 20)	conv2	N4(32, 128, 12, 20)	128	5 × 5	1
N4(32, 128, 12, 20)	pool2	N5(32, 128, 6, 10)	128	2 × 2	2
N5(32, 128, 6, 10)	conv3	N6(32, 128, 6, 10)	128	5 × 5	1
N6(32, 128, 6, 10)	conv4	N7(32, 64, 6, 10)	64	5 × 5	1
N7(32, 64, 6, 10)	dropout	N7(32, 64, 6, 10)	64	-	-
N7(32, 64, 6, 10)	pool3	N8(32, 64, 3, 5)	64	2 × 2	2
N8(32, 64, 3, 5)	flatten	N9(32, 960)	960	-	-
N9(32, 960)	fc	N10(32, 4)	4	-	-

**Table 2:** Channel and frequency attention (CFA) module’s network structure.

Input	Layer	Output	Feature Maps
(32, 177, 60, 100)	AdaptiveMaxPool2d	(32, 177, 1, 1)	177
(32, 177, 60, 100)	AdaptiveAvgPool2d	(32, 177, 1, 1)	177
(32, 177, 1, 1)	Linear(fc1)	(32, 59)	59
(32, 177, 1, 1)	ReLU	(32, 59)	59
(32, 59)	Linear(fc2)	(32, 177)	177
(32, 177)	ReLU	(32, 177)	177
(32, 177)	Sigmoid	(32, 177, 1, 1)	177

**Table 3:** Classification results of TF-CNN-CFA model in 7 subjects (3 s).

Subject	Accuracy	Precision	Recall	*F*-Score	Kappa
S1	0.8661	0.8724	0.8661	0.8663	0.8214
S2	0.7304	0.7488	0.7304	0.7263	0.6406
S3	0.6670	0.7309	0.6670	0.6555	0.5569
S4	0.7116	0.7505	0.7116	0.7051	0.6151
S5	0.6920	0.7280	0.6920	0.6854	0.5890
S6	0.7536	0.7645	0.7536	0.7524	0.6715
S7	0.9089	0.9151	0.9089	0.9091	0.8784
Mean	0.7614	0.7872	0.7614	0.7572	0.6818
Std.	0.0913	0.0749	0.0913	0.0950	0.1215

**Table 4:** A comparison of the different models on the 3000 ms data (five-fold cross-validation). The best results for each evaluation metric are highlighted in bold. The first three network models take raw signals as input, while the following five models take time–frequency maps as input after feature representation.

Model	Accuracy	Precision	Recall	*F*-Score	Kappa	AUC Value
EEGNet_4_2	0.27 ± 0.02	0.27 ± 0.02	0.27 ± 0.02	0.26 ± 0.02	0.02 ± 0.03	0.51 ± 0.01
EEGNet_8_2	0.26 ± 0.03	0.26 ± 0.03	0.26 ± 0.03	0.26 ± 0.02	0.02 ± 0.03	0.51 ± 0.02
EEGNex	0.36 ± 0.08	0.35 ± 0.08	0.36 ± 0.08	0.35 ± 0.08	0.14 ± 0.10	0.57 ± 0.05
TF-ResNet18	0.22 ± 0.01	0.06 ± 0.01	0.22 ± 0.01	0.09 ± 0.01	0.00 ± 0.01	0.50 ± 0.00
TF-VGG16	0.25 ± 0.00	0.06 ± 0.00	0.25 ± 0.00	0.10 ± 0.00	0.00 ± 0.00	0.50 ± 0.00
TF-LeNet	0.43 ± 0.08	0.49 ± 0.09	0.43 ± 0.08	0.38 ± 0.11	0.25 ± 0.11	0.62 ± 0.05
**TF-CNN**	**0.70 ± 0.08**	**0.76 ± 0.07**	**0.70 ± 0.08**	**0.69 ± 0.08**	**0.60 ± 0.10**	**0.80 ± 0.05**
**TF-CNN-CFA**	**0.76 ± 0.09**	**0.79 ± 0.07**	**0.76 ± 0.09**	**0.76 ± 0.10**	**0.68 ± 0.12**	**0.84 ± 0.06**

**Table 5:** Performance comparison of the four best neural network architectures on the self-collected dataset (3000 ms). The best accuracy and Kappa coefficient values for each subject are highlighted in bold.

Subjects	EEGNex		TF-LeNet		TF-CNN		TF-CNN-CFA	
	Acc	Kappa	Acc	Kappa	Acc	Kappa	Acc	Kappa
S1	0.3679 ±0.03	0.1578 ±0.04	0.5232 ±0.09	0.3638 ±0.09	0.8295 ±0.05	0.7724 ±0.08	**0.8661** **±0.02**	**0.8214** **±0.03**
S2	0.3598 ±0.03	0.1475 ±0.04	0.3920 ±0.10	0.1926 ±0.12	0.6134 ±0.15	0.4838 ±0.17	**0.7304** **±0.05**	**0.6406** **±0.04**
S3	0.3259 ±0.02	0.1003 ±0.03	0.4589 ±0.06	0.2783 ±0.07	0.6643 ±0.07	0.5520 ±0.09	**0.6670** **±0.08**	**0.5569** **±0.12**
S4	0.4545 ±0.05	0.2730 ±0.06	0.3143 ±0.05	0.0931 ±0.06	**0.7366** **±0.04**	**0.6491** **±0.05**	0.7116 ±0.03	0.6151 ±0.05
S5	0.3223 ±0.02	0.0959 ±0.02	0.3464 ±0.08	0.1353 ±0.11	0.6152 ±0.09	0.4872 ±0.12	**0.6920** **±0.04**	**0.5890** **±0.05**
S6	0.2295 ±0.02	0.0285 ±0.04	0.4821 ±0.07	0.3096 ±0.08	0.7196 ±0.05	0.6266 ±0.05	**0.7536** **±0.04**	**0.6715** **±0.04**
S7	0.4366 ±0.06	0.2489 ±0.08	0.5196 ±0.08	0.3585 ±0.10	0.7250 ±0.13	0.6340 ±0.17	**0.9089** **±0.03**	**0.8784** **±0.03**
Mean	0.3566 ±0.08	0.1421 ±0.10	0.4338 ±0.08	0.2473 ±0.10	0.7005 ±0.08	0.6007 ±0.10	0.7614 ±0.098	0.6818 ±0.12

**Table 6:** The number of training and testing samples obtained by segmenting the original measurement data into different non-overlapping lengths.

N. Points	Training Set	Test Set	Image Resolution
1.5 s	13,440	3360	60 × 100
2.0 s	10,080	2520	60 × 100
2.5 s	8064	2016	60 × 100
3.0 s	6720	1680	60 × 100
3.5 s	5712	1428	60 × 100
4.0 s	5040	1260	60 × 100
4.5 s	4436	1108	60 × 100
5.0 s	4032	1008	60 × 100

**Table 7:** The validation accuracy based on different network models (per category and overall performance), utilizing K-fold cross-validation, with K = 5 folds. The best overall accuracy for each time length *L* is highlighted in bold.

Time Lengths *L*	Model	Class 1	Class 2	Class 3	Class 4	Overall
5.0 s	TF-LeNet	0.39	0.34	0.33	0.42	0.37 ± 0.04
	TF-CNN	0.52	0.61	0.59	0.54	0.57 ± 0.04
	TF-CNN-CFA	0.65	0.56	0.65	0.58	**0.61 ± 0.05**
4.5 s	TF-LeNet	0.31	0.37	0.36	0.48	0.38 ± 0.07
	TF-CNN	0.57	0.59	0.5	0.48	0.54 ± 0.05
	TF-CNN-CFA	0.63	0.63	0.71	0.55	**0.63 ± 0.07**
4.0 s	TF-LeNet	0.42	0.42	0.32	0.48	0.41 ± 0.07
	TF-CNN	0.69	0.62	0.61	0.58	0.63 ± 0.05
	TF-CNN-CFA	0.75	0.66	0.68	0.66	**0.69 ± 0.04**
3.5 s	TF-LeNet	0.44	0.4	0.38	0.41	0.41 ± 0.03
	TF-CNN	0.66	0.7	0.67	0.67	0.68 ± 0.02
	TF-CNN-CFA	0.8	0.72	0.65	0.68	**0.71 ± 0.07**
3.0 s	TF-LeNet	0.51	0.39	0.45	0.39	0.44 ± 0.06
	TF-CNN	0.67	0.74	0.71	0.69	0.7 ± 0.03
	TF-CNN-CFA	0.8	0.72	0.77	0.75	**0.76 ± 0.03**
2.5 s	TF-LeNet	0.57	0.46	0.55	0.53	0.53 ± 0.05
	TF-CNN	0.8	0.69	0.73	0.67	0.72 ± 0.06
	TF-CNN-CFA	0.79	0.75	0.73	0.73	**0.75 ± 0.03**
2.0 s	TF-LeNet	0.64	0.52	0.45	0.45	0.52 ± 0.09
	TF-CNN	0.77	0.8	0.78	0.78	0.78 ± 0.01
	TF-CNN-CFA	0.84	0.83	0.85	0.79	**0.83 ± 0.03**
1.5 s	TF-LeNet	0.64	0.52	0.57	0.65	0.6 ± 0.06
	TF-CNN	0.89	0.83	0.86	0.82	0.85 ± 0.03
	TF-CNN-CFA	0.92	0.86	0.87	0.83	**0.87 ± 0.04**

## Data Availability

The data presented in this study are available on request from the corresponding author. The data are not publicly available due to privacy and ethical restrictions.
